# Efficacy and safety of FOLFIRI/aflibercept (FA) in an elderly population with metastatic colorectal cancer (mCRC) after failure of an oxaliplatin-based regimen

**DOI:** 10.1371/journal.pone.0269399

**Published:** 2022-06-03

**Authors:** Nieves Martínez-Lago, Soledad Cameselle García, Beatriz Alonso de Castro, Martín I. Gómez-Randulfe Rodríguez, Marta Carmona Campos, Paula González Villarroel, Mercedes Salgado Fernández, Juan C. De la Cámara Gómez, Carlos Romero Reinoso, Antía Cousillas Castiñeiras, José Carlos Méndez Méndez, Yolanda Vidal Insua, Ana Fernández-Montes

**Affiliations:** 1 Medical Oncology Department, University Hospital A Coruña, A Coruña, Spain; 2 Medical Oncology Department, Ourense University Hospital Complex, Ourense, Spain; 3 Medical Oncology Department, University Hospital Lucus Augusti, Lugo, Spain; 4 Medical Oncology Department, Alvaro Cunqueiro University Hospital, Vigo, Spain; 5 Medical Oncology Department, POVISA, Vigo, Spain; 6 Medical Oncology Department, Pontevedra University Hospital Complex, Pontevedra, Vigo, Spain; 7 Medical Oncology Department, Centro Oncológico de Galicia, A Coruña, Spain; 8 Medical Oncology Department, Santiago University Hospital Complex, Santiago de Compostela, A Coruña, Spain; University of Nebraska Medical Center, UNITED STATES

## Abstract

**Background:**

The VELOUR study showed the benefit of FOLFIRI-Aflibercept (FA) versus FOLFIRI in patients with metastatic colorectal cancer (mCRC) in second-line treatment. However, only 36% of the included patients were ≥65 years. Thus, we seek to evaluate the efficacy and safety of FA in the elderly population in the context of routine practice.

**Materials and methods:**

We conducted an observational, retrospective, multicenter, observational study of patients ≥70 years with mCRC treated with FA after progression to oxaliplatin chemotherapy in routine clinical practice in 9 hospitals of the GITuD group.

**Results:**

Of 388 patients treated with FA between June 2013 and November 2018, 75 patients ≥70 years were included. The median number of cycles was 10 and the objective response (ORR) and disease control rates (DCR) were 33.8% and 72.0%, respectively. With a median follow-up of 27.1 months, median Progression-free survival (PFS) was 6.6 months and median Overall Survival (OS) was 15.1 months. One third fewer metastasectomies were performed in the ≥75 years’ subgroup (24 vs. 52%, p = 0.024) and more initial FOLFIRI dose reductions (68 vs. 36%, p = 0.014). ORR (23.8% vs. 38.3%), DCR (42.8% vs. 85.1%), and PFS (4 vs. 7.8 months; p = 0.017) were significantly less, without difference in OS (9.9 vs. 17.1 months; p = 0.129). The presence of prior hypertension (HT) (PFS 7.9 vs. 5.7 months, p = 0.049) and HT ≥ grade 3 during treatment (PFS 7.6 vs. 6.6 months, p = 0.024) were associated with longer PFS. The most frequent grade 3/4 adverse events were: asthenia (21.3%), neutropenia (14.7%), and diarrhea (14.7%). 57.3% required FOLFIRI dose reduction; 34.7% of aflibercept, including discontinuation (5.3% and 18.7%, respectively).

**Conclusions:**

FA combination is effective in patients ≥70 years. The occurrence of HT is predictive of efficacy. Close monitoring of toxicity and initial dose adjustment is recommended.

## Introduction

Colorectal cancer (CRC) is the second most common neoplasm in Europe and the number one cause of cancer worldwide, accounting for 935,000 deaths in 2020 [1]. Age represents one of the leading risk factors for developing CRC, with an incidence ranging from 68 cases per 100,000 inhabitants aged 55–59 years to 258.8 per 100,000 for those ≥85 years of age [2]. Similarly, age impacts prognosis with an OS rate of 69% for those <65 years versus 62% for those ≥65 years, which would reflect a different biology of CRC based on age at diagnosis. Thus, in elderly patients, a greater tendency of right colon presentation has been identified, at the expense of a decrease in rectal tumors and, to a lesser extent, tumors of the left colon, especially in individuals ≥80 years of age [2].

The treatment of mCRC is based on the combination of polychemotherapy schemes based on oxaliplatin and/or irinotecan associated with biological agents, such as anti-EGFR or antiangiogenic agents, depending on parameters such as the mutational status of RAS and BRAF or tumor sidedness [3].

Aflibercept is a recombinant fusion protein, formed by portions of the VEGF binding sites of the extracellular domains of the human VEGFR-1 and 2 receptors, fused to an Fc portion of human IgG1 [4]. Its mechanism of action is known as “VEGF trapping” and consists of binding to VEGF-A, VEGF-B, and PIGF, thereby preventing the binding of these soluble factors to their endogenous receptors with its consequent promotion of angiogenesis.

The benefit of aflibercept (vs. placebo) combined with FOLFIRI following failure of an oxaliplatin-based chemotherapy regimen, defined as progression to a first line of oxaliplatin-based chemotherapy, was evidenced in the VELOUR study, a randomized, multicenter, phase III study with significantly increased OS 13. 5 vs. 12.1 months (HR 0.817; 95% CI 0.71–0.94; p = 0.0032), PFS 6.9 vs. 4.7 months (HR 0.76 95% CI, 0.66–0.87) and ORR 19.8 vs. 11.1% (p = 0.0001) [5]. These results were key for FOLFIRI and aflibercept being approved as second line mCRC treatment by the various regulatory agencies.

However, the elderly population, defined as patients ≥65 years of age, represented only 36% of the entire VELOUR study population [6]. A *post hoc* analysis of this study, which analyzed the efficacy and safety of FA in different age subgroups, compared the elderly versus participants <65 years of age and found no significant differences in terms of toxicity or efficacy [6]. Thus, the OS benefit was seen to remain both in the ≥65 years (12.6 vs. 11.3 months; HR 0.85; 95% CI 0.68–1.07) and <65 years (14.5 vs. 12.5 months; HR 0.80; 95% CI 0.67–0.95) strata. Adverse events were comparable in subjects regardless of age. The incidence of grade 3/4 adverse events related to antiangiogenic therapy was higher in the ≥65 years’ subgroup, in both the aflibercept (89.3% vs. 80.5%) and placebo arms (67.4% vs. 59.4%).

As mentioned, the elderly population is underrepresented in randomized clinical trials, which are often limited to small, homogeneous groups of patients in specialized, controlled settings. Observational studies are needed to enable us to evaluate drug efficacy and safety in routine clinical practice [7]; consequently, the aim of our study is to evaluate efficacy and safety in FOLFIRI-aflibercept therapy in people ≥70 years of age in the context of routine clinical practice, as well as to perform an exploratory analysis of various predictive and/ or prognostic factors, including different age subgroups.

## Materials and methods

### 2.1 | Study design

We conducted an observational, retrospective, multicenter study in elderly patients, defined as ≥70 years, with mCRC treated with FA after progression following first line with oxaliplatin-based therapy, or after a period of <6 months from the end of oxaliplatin-based adjuvant treatment (called, ‘rapid progressors’), in 9 Galician hospitals belonging to the Galician Group of Research in Digestive Tumors (GITuD). The study was approved by the local ethic committee and was conducted in accordance with the Declaration of Helsinki. All participants gave their written informed consent prior to inclusion in the study.

For inclusion in the study, patients had to have been treated with FA in the context of routine clinical practice. The full dose of FA included: aflibercept 4 mg/kg intravenously (IV) over 1 hour, followed by FOLFIRI (irinotecan 180 mg/m2 IV over 90 minutes, with leucovorin 400 mg/m2 IV over 2 hours, followed by FU 400 mg/m2 bolus and FU 2400 mg/m2 continuous infusion over 46 hours). The treatment cycle was repeated every 2 weeks. Patients with aflibercept initial doses reduced (2 mg/kg) or FOLFIRI initial doses reduced (80% or 60% of full dose) were included. Those who had previously received irinotecan, antiangiogenic agents other than aflibercept, or chemotherapies other than FOLFIRI in combination with aflibercept were excluded.

### 2.2 | Data collection

Clinicopathological and treatment data were collected from subjects’ medical records including gender, age, performance status (ECOG PS), and history of hypertension (HT). Disease characteristics included RAS and BRAF mutational status, mismatch repair proteins expression (MMR), primary tumor location, histological grade, synchronous or metachronous tumor presentation, resection of primary tumor, resection of metastatic disease, and previous treatment received. The initial dose of FA, number of cycles administered, response obtained, dose delays and/ or dose reductions, toxicity according to CTCAE 4.0 criteria, and progression and/or survival were collected and analyzed retrospectively.

### 2.3 | Statistical analyses

OS was defined as the time between treatment initiation and all-cause mortality. PFS was defined as the interval between treatment initiation and radiological confirmation of disease progression or death from any cause. ORR was defined as the proportion of cases who achieved a partial (PR) or complete response (CR), and DCR as the proportion of subjects who achieved CR, PR or SD of at least six weeks after treatment initiation. Toxicity data were collected according to the NCI-CTCAE v4.0.

Statistical analyses were performed using SPPS v25.0. Chi-square test or Fisher’s exact test (depending on sample size) was used to compare clinical and demographic variables. The Kaplan-Meier model was applied to estimate median PFS and OS and 95% confidence intervals (CI). Differences between survival curves were compared by log-rank test with a bilateral significance of 0.05.

## Results

### Population characteristics

Of 338 individuals treated with FA between June 2013 and November 2018, 75 elderly patients were included in this study. Patient characteristics are summarized in Table 1. The median age was 72.7 years (range 70–84 years), with 33.3% ≥75 years. All had previously received an oxaliplatin-based regimen, both in adjuvant (5.3%) and first-line advanced disease (94.7%).

**Table 1:** Study population characteristics.

Characteristics	N = 75 (%)
**Age**	
**Median (range)**	72.7 years (70–84 years)
** 70–75 years**	50 (66.7%)
** 75–80 years**	20 (26.7%)
** >80 years**	5 (6.6%)
**Gender**	
**Male**	49 (65.3%)
**Female**	26 (34.7%)
**ECOG PS**	
**0–1**	63 (84.0%)
**2–3**	9 (12.0%)
**Unknown**	3 (4.0%)
**Tumor site**	
**Right-sided**	27 (36.0%)
**Left-sided**	31 (41.3%)
**Rectum**	15 (20.0%)
**Unknown**	2 (2.7%)
**Histological Grade**	
**Low grade (G1-G2)**	57 (76.0%)
**High grade (G3)**	8 (10.7%)
**Unknown**	10 (13.3%)
**RAS/BRAF status**	
**RAS/BRAFwt**	26 (34.7%)
**RASmt**	47 (62.7%)
**BRAFmt**	1 (1.3%)
**Unknown**	1 (1.3%)
**Mismatch Repair Proteins (N = 23)**	
**Conserved**	22 (95.7%)
**Deficiency**	1 (4.3%)
**Tumor Presentation**	
**Synchronous**	51 (68.0%)
**Metachronous**	24 (32.0%)
**Primary Tumor Surgery**	
**No**	17 (22.7%)
**Yes**	58 (77.3%)
**Metastasectomy**	
**No**	43 (57.3%)
**Yes**	32 (42.7%)
**Prior therapy**	
**Adjuvant CT**	4 (5.3%)
**FOLFOX**	9 (12.0%)
**FOLFOX/CAPOX-BEV**	44 (58.7%)
**FOLFOX-antiEGFR**	18 (24.0%)

ECOG PS Eastern Cooperative Oncology Group performance status, CT Chemotherapy, BEV Bevacizumab

### Efficacy

Patients received a median of 10 cycles of FA (range 1–35 cycles). Of the 75 participants included, 68 were evaluable for response. The ORR was 33.8%, all of which were partial responses, and DCR was 72% (Table 2).

**Table 2:** Response rate.

Response	N = 68 (%)
**Complete Response (CR)**	0 (0.0)
**Partial Response (PR)**	23 (33.8)
**Stable Disease (SD)**	26 (38.2)
**Disease Progression (PD)**	19 (27.9)
**ORR (CR+PR)**	**23 (33.8)**
**DCR (CR+PR+SD)**	**49 (72.0)**

**Fig 1:**
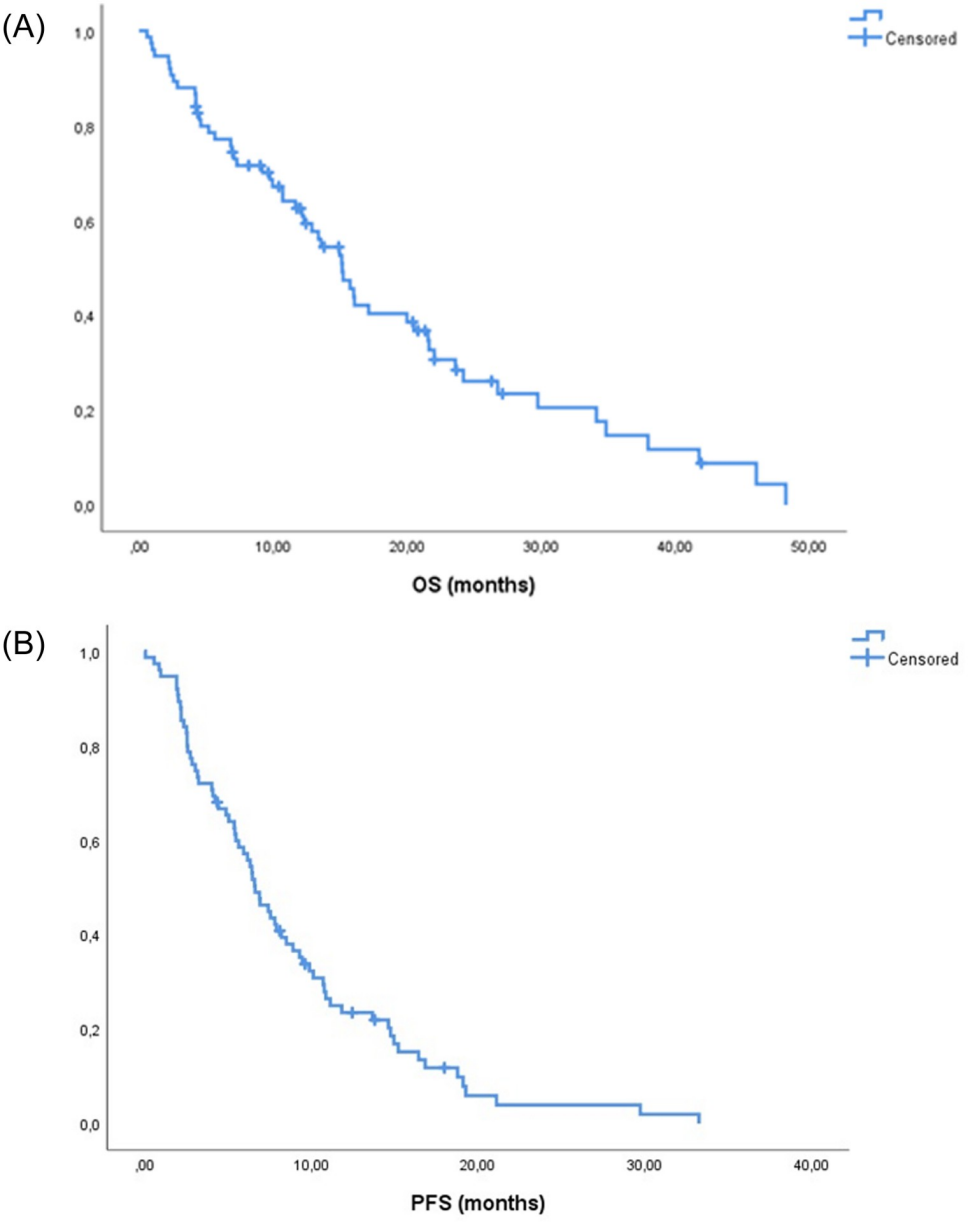
Kaplan-Meier curves for Overall Survival (A) and Progression-Free Survival (B).

With a median follow-up of 27.1 months, median PFS was 6.6 months (95% CI, 5.3–7.9 months) and median OS was 15.1 months (95% CI, 12.5–17.8 months) (Fig 1).

### Safety

The most common adverse events are presented in Table 3. The most frequent toxicities were asthenia (72.0%), mucositis (49.3%), diarrhea (48%), neutropenia (48%), and anemia (44%). As for grade3/4 toxicities, asthenia (21.3%), neutropenia (14.7%), and diarrhea (14.7%) were the most frequent. Two patients developed grade 5 toxicity, including one cerebrovascular event and one intestinal perforation.

**Table 3:** Summary of the most common all grade and grade 3/4 adverse events.

Toxicity	All Grades	Grade 3/4
**Neutropenia**	36 (48.0%)	11 (14.7%)
**Anemia**	33 (44.0%)	4 (5.3%)
**Thrombopenia**	24 (32.0%)	4 (5.3%)
**Nausea/vomiting**	27 (36.0%)	1 (1.3%)
**Diarrhea**	36 (48.0%)	11 (14.7%)
**Stomatitis**	37 (49.3%)	7 (9.3%)
**Asthenia**	54 (72.0%)	16 (21.3%)
**Hypertension**	19 (25.3%)	4 (5.3%)
**Proteinuria**	12 (16.0%)	2 (2.7%)
**Dysphonia**	16 (21.3%)	4 (5.3%)
**VTE**	11 (14.7%)	----

Toxicity was managed by reducing the dose of Aflibercept dose in 34.7% of patients, including discontinuation in 18.7%, while 57.3% required a reducing the dose of FOLFIRI, and treatment was discontinued in 5.3% (Table 4).

**Table 4:** Dose reduction and discontinuation during FOLFIRI aflibercept treatment.

**FOLFIRI initial dose**	
Full dosage	40 (53.3)
80%	32 (42.7)
60%	3 (4.0)
**Aflibercept initial dose**	
4 mg/kg	72 (96.0)
2 mg/kg	3 (4.0)
**FOLFIRI dose reduction**	
No	32 (42.7)
Level -1 (80%)	32 (42.7)
Level -2 (60%)	7 (9.3)
Discontinuation	4 (5.3)
**Aflibercept dose reduction**	
No	49 (65.3)
2 mg/kg	12 (16.0)
Discontinuation	14 (18.7)

### Prognostic and predictive factors

Exploratory analyses were performed to identify predictive factors for PFS and OS (Table 5). Findings included significantly higher PFS in the <75 years’ subgroup (7.8 vs. 4.1 months, p = 0.015) and a non-significant trend in OS (17.1 vs. 10 months, p = 0.129). Likewise, tumor location in the left vs. right colon and rectum has also proven to affect OS (20.5 vs. 12.9 vs. 9.8 months, p = 0.021). Synchronous tumor presentation has been associated with worse prognosis, both in terms of PFS (6.3 vs. 8.2 months, p = 0.031) and OS (12.9 vs. 21.6 months, p = 0.048). Primary tumor resection has also exhibited a prognostic effect on both PFS (6.9 vs. 4.9 months, p = 0.049) and OS (16 vs. 9.8 months, p = 0.020). Finally, the absence of repair protein expression (dMMR) significantly impacted PFS (1 vs. 10.8 months, p = 0.001) and OS (1 vs. 21.6 months, p = 0.001). No significant differences were detected according to sex, ECOG PS, histologic grade, metastasectomies, or RAS or BRAF mutational status.

**Table 5:** Prognostic factors.

Characteristics	PFS (months)	p-value	OS (months)	p-value
**Age**				
<75 years	7.8	0.015	17.1	0.129
≥75 years	4.1		10.0	
**Sex**				
Male	6.6	0.868	16.0	0.073
Female	6.6		12.9	
**ECOG PS**				
0–1	6.9	0.160	15.7	0.962
2–3	6.6		15.1	
**Previous HT**				
No	5.7	0.049	15.7	0.415
Yes	7.9		15.1	
**Tumor location**				
Right-sided	5.5	0.198	12.9	0.021
Left-sided	8.9		20.5	
Rectum	5.7		9.2	
**Histological grade**				
Low grade	7.6	0.472	15.7	0.135
High grade	4.1		6.8	
**RAS/BRAF status**				
RAS/BRAFwt	6.9	0.472	16.0	0.472
RASmt	6.3		15.1	
BRAFmt	3.3		7.1	
**Mismatch Repair Protein expression (n = 23)**				
Conserved	10.8	0.001	21.6	0.001
Deficiency	1.0		1.0	
**T. presentation**				
Synchronous	6.3	0.031	12.9	0.048
Metachronous	8.2		21.6	
**Primary tumor surgery**				
No	4.9	0.0498	9.8	0.020
Yes	6.9		16.0	
**Metastasectomy**				
No	6.3	0.246	12.3	0.103
Yes	7.4		16.1	

A positive history of hypertension (HT) was significantly associated with longer PFS: 7.9 vs. 5.7 months (p = 0.049). Likewise, the appearance of HT ≥ grade 3 during FA treatment identified a population with better PFS: 7.6 vs. 6.6 months (p = 0.024). In contrast, those receiving full dose FOLFIRI exhibited a trend toward significantly longer PFS (7.4 vs. 6.3 months, p = 0.098) and OS: 21.7 vs. 10.6 months (p = 0.001) (Table 6). No significant association was observed in survival as a function of the dose of aflibercept administered or the digestive and hematologic toxicity developed during FA treatment.

**Table 6:** Predictive factors.

Characteristics	PFS (months)	p-value	OS (months)	p-value
**Previous HTA**				
**No**	5.7	0.049	15.7	0.415
**Yes**	7.9		15.1	
**FOLFIRI initial dose**				
**Full**	7.4	0.098	21.6	0.001
**Reduced**	6.3		10.7	
**Aflibercept initial dose**				
**Full**	6.6	0.974	15.1	0.830
**Reduced**	9.4		29.7	
**Neutropenia**				
**G0-G2**	6.4	0.179	15.1	0.255
**G3-G4**	7.9		26.7	
**Anemia**				
**G0-G2**	6.6	0.153	15.1	0.919
**G3-G4**	9.9		9.8	
**Thrombopenia**				
**G0-G2**	6.9	0.881	15.1	0.928
**G3-G4**	1.9		2.3	
**Asthenia**				
**G0-G2**	6.9	0.852	15.2	0.573
**G3-G4**	4.9		15.1	
**Nausea/Vomiting**				
**G0-G2**	6.6	0.694	15.1	0.834
**G3-G4**	6.6		16.0	
**Diarrhea**				
**G0-G2**	6.9	0.852	15.1	0.357
**G3-G4**	6.6		13.6	
**Stomatitis**				
**G0-G2**	6.5	0.694	15.1	0.669
**G3-G4**	11.0		22.0	
**Hypertension**				
**G0-G2**	6.6	0.024	15.1	0.496
**G3-G4**	7.6		34.1	
**Proteinuria**				
**G0-G2**	6.6	0.494	15.2	0.188
**G3-G4**	2.8		4.3	
**Dysphonia**				
**G0-G2**	6.5	0.269	15.0	0.876
**G3-G4**	10.9		16.0	

### Age subgroups analysis

An exploratory analysis was performed by age subgroup (70–75 vs. ≥75 years) and is detailed in Table 7. Fewer participants ≥75 years underwent metastasectomies (24 vs. 52%, p = 0.024); likewise, there was a trend towards a higher proportion of patients with the tumor located in the right colon (52 vs. 28%, p = 0.065). As for FA treatment, a higher percentage of patients with reduced-dose FOLFIRI initiation (68 vs. 36%, p = 0.014) was identified in the older subgroup, with no differences regarding aflibercept (96% vs. 96%, p = 0.990).

**Table 7:** Population characteristics according to age subgroups.

	Global (N = 75)	70–75 years (N = 50)	>75 years (N = 25)	P
**Sex**				
Male	49 (65.3%)	29 (58.0%)	20 (80.0%)	0.074
Female	26 (34.7%)	21 (42.0%)	5 (20.0%)	
**ECOG-PS**				
0–1	63 (84.0%)	42 (84.0%)	21 (84.0%)	0.710
2–3	9 (12.0%)	5 (10.0%)	4 (16.0%)	
Unknown	3 (4.0%)	3 (6.0%)	0 (0.0%)	
**Tumor location**				
Right Sided	27 (36.0%)	14 (28.0%)	13 (52.0%)	0.065
Left Sided	31 (41.3%)	25 (50.0%)	6 (24.0%)	
Rectum	15 (20.0%)	9 (18.0%)	6 (24.0%)	
Unknown	2 (2.7%)	2 (4.0%)		
**Histological grade**				
Low grade (G1-G2)	57 (76.0%)	38 (76.0%)	19 (76.0%)	0.999
High grade (G3)	8 (10.7%)	5 (10.0%)	3 (12.0%)	
Unknown	10 (13.3%)	7 (14.0%)	3 (12.0%)	
**RAS/BRAF status**				
RAS/BRAFwt	26 (34.7%)	18 (36.0%)	8 (32.0%)	0.999
RASmt	47 (62.7%)	31 (62.0%)	16 (64.0%)	
BRAFmt	1 (1.3%)	1 (2.0%)	1 (4.0%)	
Unknown	1 (1.3%)	0 (0.0%)	1 (4.0%)	
**Tumor presentation**				
Synchronous	51 (68%)	33 (66.0%)	18 (72.0%)	0.793
Metachronous	24 (32%)	17 (34.0%)	7 (28.0%)	
**Primary tumor surgery**				
No	17 (22.7%)	9 (18.0%)	8 (32.0%)	0.2424
Yes	58 (77.3%)	41 (82.0%)	17 (68.0%)	
**Metastasectomy**				
No	43 (57.3%)	24 (48.0%)	19 (76.0%)	0.027
Yes	32 (42.7%)	26 (52.0%)	6 (24.0%)	
**FOLFIRI initial dose**				
Full	40 (53.3%)	32 (64.0%)	8 (32.0%)	0.014
Reduced	35 (46.7%)	18 (36.0%)	17 (68.0%)	
**Aflibercept initial dose**				
Full	72 (96.0%)	48 (96.0%)	24 (96.0%)	0.990
Reduced	3 (4.0%)	2 (4.0%)	1 (4.0%)	

**Fig 2:**
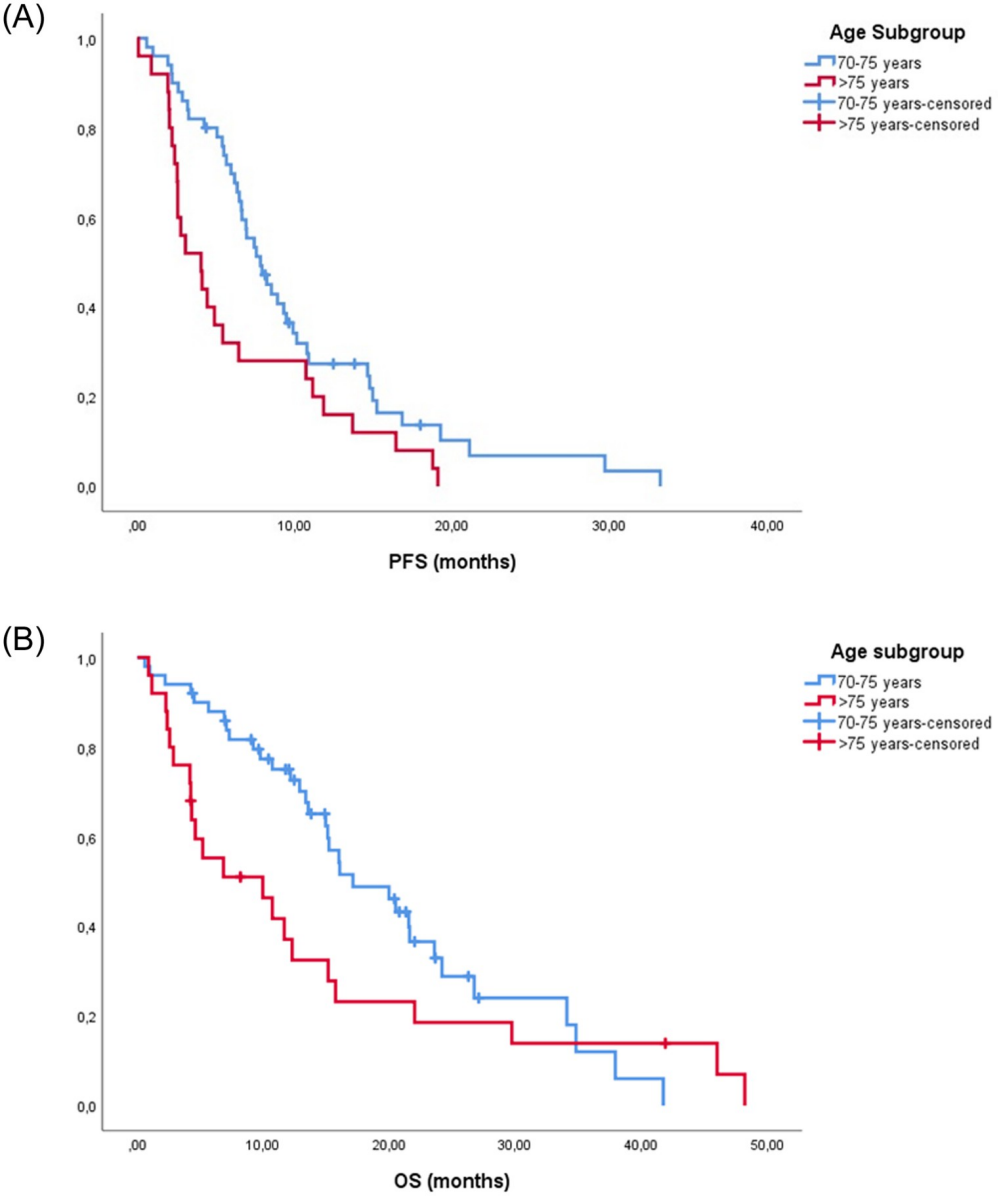
Kaplan-Meier curves according to age subgroups for Progression-Free Survival (A) and Overall Survival (B).

Patients ≥75 years was associated with significantly lower ORR (23.8% vs. 38.3%) and DCR (42.8% vs. 85.1%) (p = 0.002). Similarly, elderly patients had a significantly lower PFS rate with a median PFS 4 vs. 7.8 months (HR 0.545, 95% CI 0.3–0.9, p = 0.017) and a non-significant trend toward shorter OS, specifically, median OS 9.9 vs. 17.1 months (HR 0.645, 95% CI 0.4–1.1, p = 0.129) (Fig 2).

In relation to adverse events, a significant increase in grade 3/4 asthenia was observed in the >75 years of age population (36.0% vs. 14.0%, p = 0.038). No significant differences were found between the remaining adverse events and the different age subgroups (Table 8).

**Table 8:** Grade 3/4 adverse events according to age subgroups.

Toxicity	Global Grade 3/4	70–75 years Grade 3/4	>75 years Grade 3/4	p
**Neutropenia**	11 (14.7%)	8 (16.0%)	3 (12%)	0.742
**Anemia**	4 (5.3%)	3 (6.0%)	1 (4.0%)	0.999
**Thrombopenia**	4 (5.3%)	3 (6.0%)	1 (4.0%)	0.999
**Nausea/vomiting**	1 (1.3%)	1 (2.0%)	0 (0.0%)	0.999
**Diarrhea**	11 (14.7%)	8 (16.0%)	3 (12%)	0.742
**Stomatitis**	7 (9.3%)	4 (8.0%)	3 (12%)	0.680
**Asthenia**	**16 (21.3%)**	**7 (14.0%)**	**9 (36.0%)**	**0.038**
**Hypertension**	4 (5.3%)	4 (8.0%)	0 (0.0%)	0.294
**Proteinuria**	2 (2.7%)	1 (2.0%)	1 (4.0%)	0.999
**Dysphonia**	4 (5.3%)	3 (6.0%)	1 (4.0%)	0.999

## Discussion

Although the population ≥65 years of age represents <10% of the world’s population, it accounts for more than 50% of new cancer diagnoses [8]. In fact, the incidence and prevalence of cancer in individuals ≥70 years of age has been on the rise in recent decades [9]. However, the elderly population is underrepresented in clinical trials. Most of the available evidence on which we base our decisions is supported by clinical trials conducted in young patients, without comorbidities, and with a good functional status. More studies are needed with greater representation of this age group.

The VELOUR study evaluated the FA combination in patients with metastatic CRC previously treated with an oxaliplatin-based regimen. The group treated with this combination displayed a significant increase in OS, PFS, and ORR versus those receiving FOLFIRI [5]. Nevertheless, the ≥65 years’ population included in the study accounted for a mere 33.5% and only 5.4% were ≥75 years. A subanalysis of this study evaluated the efficacy and safety of the FA combination in patients ≥65 years [6]. No differences in OS, PFS, or toxicity were detected as a function of age (≥65 and <65 years). Nonetheless, the low percentage of elderly patients enrolled in this type of study does not allow us to reliably reflect the real situation of this population group. Therefore, there is a need for studies that allow us to adapt treatments to the population we encounter in routine clinical practice.

The OZONE study is a prospective, observational study that evaluated the efficacy and safety of FA in a real life setting [10]. This study allowed the inclusion of special populations, such as patients with liver failure and renal insufficiency, who had been excluded from the VELOUR study [5]. The elderly population was also specifically evaluated. In fact, 48% of the patients included in the OZONE study were ≥65 years, without differences in efficacy or safety in this population [10].

We have conducted a multicenter, retrospective study to evaluate the efficacy and safety of FA in the elderly population in routine clinical practice. Patients ≥70 years were included, one third of whom were ≥75 years old and 12% frail (ECOG 2–3). OS was 15.1 months and PFS was 6.6 months, comparable to the VELOUR study [5]. However, when evaluating the results by age, a decrease in PFS (7.8 vs. 4.1 months; p = 0.015) and a non-significant trend to a lower OS (17.1 vs. 10 months) were observed in patients ≥75 years.

In our study, treatment exposure was higher than in the VELOUR study; a median of 10 cycles of FA were administered. An ORR of 33.8% was achieved, higher than the 19.8% in VELOUR study [5]. The population ≥75 years included in our study exhibited a lower ORR (23.8% vs. 38.3%, p = 0.002) and also a lower median number of cycles received than the younger population. Contrary to the VELOUR study [5], almost half of the participants initiated treatment at a reduced dose. Some 46.7% of cases initiated FOLFIRI at 80%; these patients had a lower OS than those who started at a full dose (21.7 vs. 10.6 months; p = 0.001), although this could be attributable to this group’s fragility. The initial dose reduction of aflibercept did not negatively impact in survival. This initial dose adjustment led to better tolerability of FA, which was reflected in lower toxicity. The incidence of anti-VEGF related toxicities was lower than in the VELOUR study [5]. HT was 25% vs 41% in the VELOUR study [5]; diarrhea was 48% vs. 69.2%, and proteinuria was detected in only 16% vs 62.2%. However, there were 2 toxic deaths attributable to anti-VEGF. Hematologic toxicities were also lower in our study compared to the VELOUR study [5] with a clear reduction in grade 3/4 neutropenia (14.7% vs. 36.7%). This toxicity led to a reduction in the dose of aflibercept in 34.7% of patients and of FOLFIRI in up to 57%. Aflibercept had to be discontinued in 18% of the subjects. When toxicity was evaluated according to age, no significant differences were found except for asthenia, which was greater in patients >75 years of age (p = 0.038).

In our study, we also performed a subgroup analysis in search of predictive variables in this age group. Greater benefit was seen in the subgroup of patients with metachronous metastases treated with FA compared to those with synchronous metastases. In all likelihood, this has to do with a higher rate of metastasectomy of metachronous vs synchronous lesions and/ or with the presence of less aggressive disease at diagnosis. Laterality has also been established in colon cancer as a negative prognostic factor for tumors located on the right side [11, 12]. In the present study, patients with right-sided colon tumors had a lower OS than those with left-sided colon tumors (12.9 vs. 20.5 months, p = 0.021). Furthermore, patients ≥75 years presented a higher percentage of neoplasms in the right colon (52% vs. 28%) and fewer metastasectomies (24% vs. 52%), which may have resulted in the lower OS and PFS of the ≥75 years’ population. Likewise, the RAS and BRAF mutational status in the primary tumor was evaluated with no significant differences being revealed.

At present, we need to find biomarkers predictive of response for the treatments we prescribe. The current study displayed a strong correlation between the development of HT and treatment efficacy (p = 0.024). Aflibercept acts as a soluble receptor that binds to VEGF-A, PlGF, and VEGF-B, thereby preventing the binding of endogenous ligands and blocking receptor-mediated signaling by inhibiting angiogenesis, tumor growth, and the development of metastasis. This alteration of angiogenesis would produce a decrease in nitrous oxide production, with the consequent development of hypertension [13]. Based on this, HT has been postulated as a class adverse effect of anti-VEGF drugs and its correlation in terms of efficacy has already been reported in other studies. For individuals with mCRC treated with BEV in first-line, there are several retrospective studies that correlate developing grade 2 and 3 HT to increased PFS and OS [14, 15]. Consistent with our data, a recent retrospective study of patients with mCRC treated in 2^nd^-line with FOLFIRI and different antiangiogenic drugs (aflibercept/ ramucirumab/ bevacizumab) established this correlation beyond the first line [16].

To our knowledge, ours is the first study to evaluate the FA combination exclusively in an elderly population. While we obtained similar results to the VELOUR study population [5], our investigation was a real-life study that allowed us to select patients outside the ideal conditions of a clinical trial. For the first time, we evaluated the impact of an initial dose reduction on efficacy and have corroborated arterial hypertension as a predictive biomarker of efficacy. Nevertheless, the present study has some limitations given its retrospective nature and the few participants included in the study. Because of these same limitations, it was not possible to perform a comprehensive geriatric assessment of these patients at the beginning of the study.

## Conclusions

The FOLFIRI-aflibercept combination is effective in elderly patients, with the appearance of hypertension being a predictive biomarker of efficacy. However, it requires careful adjustment of the initial dose to control possible adverse effects.

## Supporting information

S1 Dataset (PDF)
