# Supplementary material for: Efficacy and safety of FOLFIRI/aflibercept (FA) in an elderly population with metastatic colorectal cancer (mCRC) after failure of an oxaliplatin-based regimen
Source: PLoS One. 2022 Jun 3;17(6):e0269399. doi: 10.1371/journal.pone.0269399 (PMC9165891; doi:10.1371/journal.pone.0269399)
Supplement: S1 Dataset — (PDF) [file pone.0269399.s001.pdf]

Minimal Data Set Definition

Statistics

EDADinicioAF

N	Valid	75
	Missing	0
Median		72,7173
Minimum		68,41
Maximum		84,27

Sexo (1:H, 2:M)

		Frequency	Percent	Valid Percent	Cumulative Percent
Valid	1	49	65,3	65,3	65,3
	2	26	34,7	34,7	100,0
	Total	75	100,0	100,0	

CodeECOG23vs01

		Frequency	Percent	Valid Percent	Cumulative Percent
Valid	,00	63	84,0	87,5	87,5
	1,00	9	12,0	12,5	100,0
	Total	72	96,0	100,0	
Missing	System	3	4,0		
Total		75	100,0		

Localización Tumor (1=derecho, 2=izquierdo, 3=recto)

		Frequency	Percent	Valid Percent	Cumulative Percent
Valid	1	27	36,0	37,0	37,0
	2	31	41,3	42,5	79,5
	3	15	20,0	20,5	100,0
	Total	73	97,3	100,0	
Missing	-99	2	2,7		
Total		75	100,0		

CODEgradoBAJOvsALTO

		Frequency	Percent	Valid Percent	Cumulative Percent
Valid	1	57	76,0	87,7	87,7
	2	8	10,7	12,3	100,0
	Total	65	86,7	100,0	
Missing	System	10	13,3		
Total		75	100,0		

CodeRAS_BRAF

		Frequency	Percent	Valid Percent	Cumulative Percent
Valid	0	26	34,7	35,1	35,1
	1	47	62,7	63,5	98,6
	2	1	1,3	1,4	100,0
	Total	74	98,7	100,0	
Missing	-99	1	1,3		
Total		75	100,0		

Exp prot reparadoras (1=conservada, 0= pérdida)

		Frequency	Percent	Valid Percent	Cumulative Percent
Valid	0	1	1,3	4,3	4,3
	1	22	29,3	95,7	100,0
	Total	23	30,7	100,0	
Missing	-99	52	69,3		
Total		75	100,0		

Presentacion (0=sincronico, 1=metacronico)

		Frequency	Percent	Valid Percent	Cumulative Percent
Valid	0	51	68,0	68,0	68,0
	1	24	32,0	32,0	100,0
	Total	75	100,0	100,0	

Cirugía Tumor 1º (0=No, 1=Si)

		Frequency	Percent	Valid Percent	Cumulative Percent
Valid	0	17	22,7	22,7	22,7
	1	58	77,3	77,3	100,0
	Total	75	100,0	100,0	

Resección M1 (1=Si, 0=N0)

		Frequency	Percent	Valid Percent	Cumulative Percent
Valid	0	43	57,3	57,3	57,3
	1	32	42,7	42,7	100,0
	Total	75	100,0	100,0	

Respuesta (0=PE, 1=EE, 2=RP, 3=RC)

		Frequency	Percent	Valid Percent	Cumulative Percent
Valid	0	19	25,3	27,9	27,9
	1	26	34,7	38,2	66,2
	2	23	30,7	33,8	100,0
	Total	68	90,7	100,0	
Missing	-99	7	9,3		
Total		75	100,0		

Means and Medians for Survival Time

Mean[a]				Median			
		95% Confidence Interval				95% Confidence Interval	
Estimate	Std. Error	Lower Bound	Upper Bound	Estimate	Std. Error	Lower Bound	Upper Bound
18,513	1,808	14,969	22,056	15,146	1,333	12,533	17,758

a. Estimation is limited to the largest survival time if it is censored.

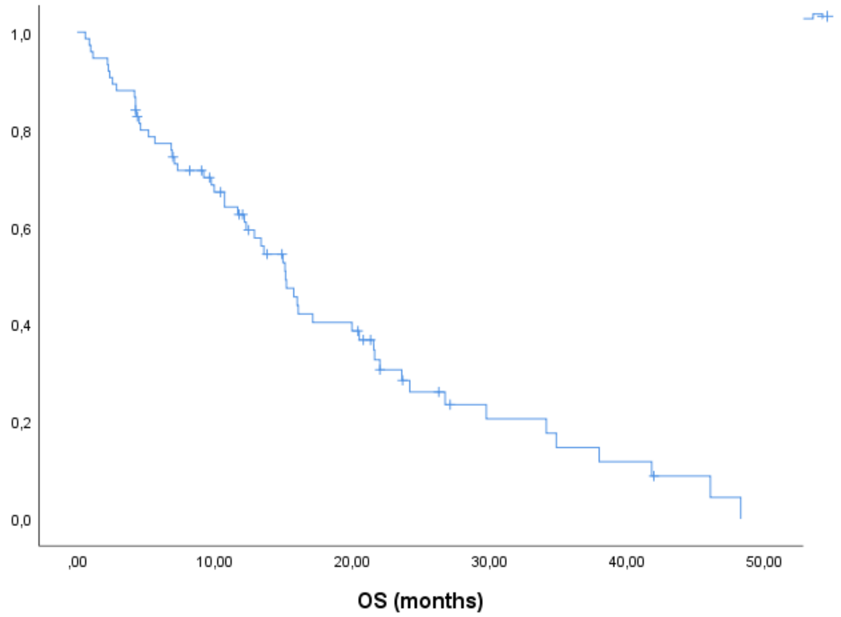

Means and Medians for Survival Time

Mean[a]				Median			
		95% Confidence Interval				95% Confidence Interval	
Estimate	Std. Error	Lower Bound	Upper Bound	Estimate	Std. Error	Lower Bound	Upper Bound
8,754	,857	7,074	10,434	6,637	,666	5,331	7,942

a. Estimation is limited to the largest survival time if it is censored.

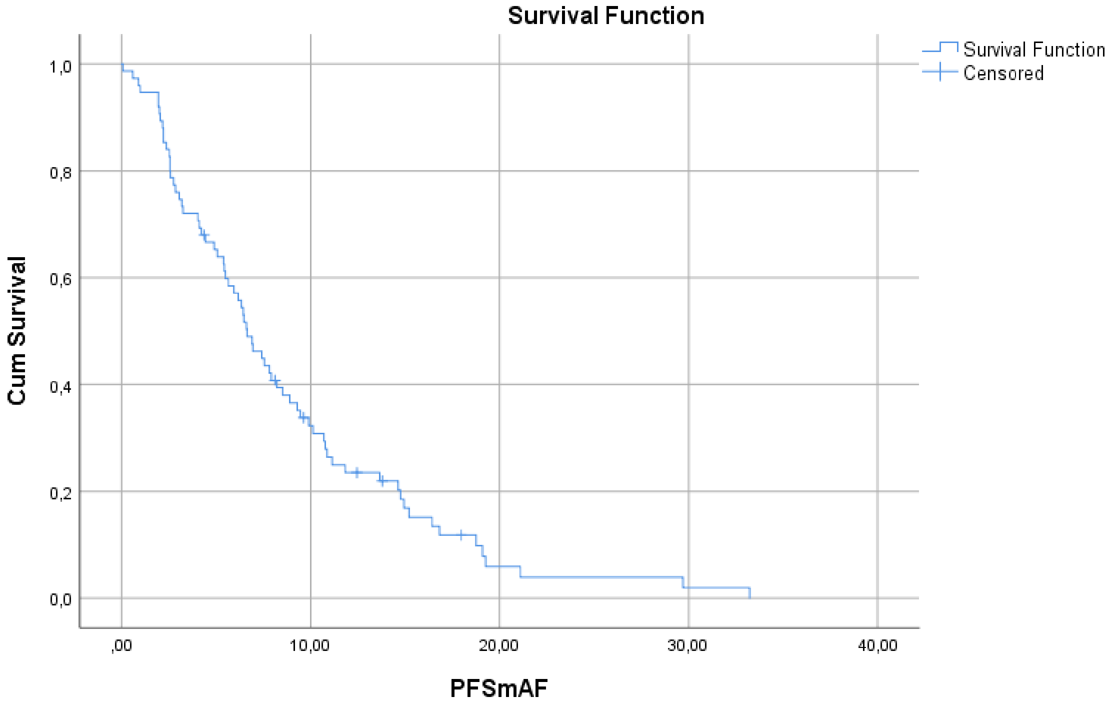

Anemia (1=G1, 2=G2, 3=G3, 4=G4)

		Frequency	Percent	Valid Percent	Cumulative Percent
Valid	0	42	56,0	56,0	56,0
	1	28	37,3	37,3	93,3
	2	1	1,3	1,3	94,7
	3	4	5,3	5,3	100,0
	Total	75	100,0	100,0	

Trombopenia (1=G1, 2=G2, 3=G3, 4=G4)

		Frequency	Percent	Valid Percent	Cumulative Percent
Valid	0	51	68,0	68,0	68,0
	1	14	18,7	18,7	86,7
	2	6	8,0	8,0	94,7
	3	4	5,3	5,3	100,0
	Total	75	100,0	100,0	

Astenia (1=G1, 2=G2, 3=G3, 4=G4)

		Frequency	Percent	Valid Percent	Cumulative Percent
Valid	0	21	28,0	28,0	28,0
	1	12	16,0	16,0	44,0

		Frequency	Percent	Valid Percent	Cumulative Percent
	2	26	34,7	34,7	78,7
	3	16	21,3	21,3	100,0
	Total	75	100,0	100,0	

Nau/Vom (1=G1, 2=G2, 3=G3, 4=G4)

		Frequency	Percent	Valid Percent	Cumulative Percent
Valid	0	48	64,0	64,0	64,0
	1	20	26,7	26,7	90,7
	2	6	8,0	8,0	98,7
	3	1	1,3	1,3	100,0
	Total	75	100,0	100,0	

Diarrea (1=G1, 2=G2, 3=G3, 4=G4)

		Frequency	Percent	Valid Percent	Cumulative Percent
Valid	0	39	52,0	52,0	52,0
	1	14	18,7	18,7	70,7
	2	11	14,7	14,7	85,3
	3	10	13,3	13,3	98,7
	4	1	1,3	1,3	100,0
	Total	75	100,0	100,0	

Mucositis (1=G1, 2=G2, 3=G3, 4=G4)

		Frequency	Percent	Valid Percent	Cumulative Percent
Valid	0	38	50,7	50,7	50,7
	1	16	21,3	21,3	72,0
	2	14	18,7	18,7	90,7
	3	7	9,3	9,3	100,0
	Total	75	100,0	100,0	

HTA (1=G1, 2=G2, 3=G3, 4=G4)

		Frequency	Percent	Valid Percent	Cumulative Percent
Valid	0	56	74,7	74,7	74,7
	1	8	10,7	10,7	85,3
	2	7	9,3	9,3	94,7
	3	4	5,3	5,3	100,0
	Total	75	100,0	100,0	

Proteinuria (1=G1, 2=G2, 3=G3, 4=G4)

		Frequency	Percent	Valid Percent	Cumulative Percent
Valid	0	63	84,0	84,0	84,0
	1	4	5,3	5,3	89,3
	2	6	8,0	8,0	97,3
	3	2	2,7	2,7	100,0
	Total	75	100,0	100,0	

Disfonía (1=G1, 2=G2, 3=G3, 4=G4)

		Frequency	Percent	Valid Percent	Cumulative Percent
Valid	0	59	78,7	78,7	78,7
	1	5	6,7	6,7	85,3
	2	7	9,3	9,3	94,7
	3	4	5,3	5,3	100,0
	Total	75	100,0	100,0	

Sangrado (1=G1, 2=G2, 3=G3, 4=G4)

		Frequency	Percent	Valid Percent	Cumulative Percent
Valid		63	84,0	84,0	84,0
	1	7	9,3	9,3	93,3
	2	4	5,3	5,3	98,7
	3	1	1,3	1,3	100,0
	Total	75	100,0	100,0	

ETEV (1=si, 0=no)

		Frequency	Percent	Valid Percent	Cumulative Percent
Valid	1	10	13,3	90,9	90,9
	4	1	1,3	9,1	100,0
	Total	11	14,7	100,0	
Missing	System	64	85,3		
Total		75	100,0		
